# Influence of Dissipated Energy on the Bonding Strength of Cold-Sprayed Titanium Coatings on Selected Metallic Substrates

**DOI:** 10.3390/ma18143355

**Published:** 2025-07-17

**Authors:** Medard Makrenek

**Affiliations:** Faculty of Management and Computer Modelling, Kielce University of Technology, 25-314 Kielce, Poland; fizmm@tu.kielce.pl

**Keywords:** cold spray, hardness, elastic modulus, adhesion, deformation work

## Abstract

Modern nanoindentation equipment allows for highly precise measurements of mechanical properties such as hardness and elastic modulus, generating detailed load–unload curves using advanced techniques and specialised software. In this study, titanium coatings were deposited on various metallic substrates using cold gas spraying. Before deposition, the spraying parameters (temperature, pressure, velocity, and distance) were statistically optimised using the Taguchi method, reducing the number of experiments required from 81 to 9. This approach allowed the identification of optimal spray conditions (T = 731.0 °C, p = 33.0 bar, V = 343.6 mm/s, d = 35.5 mm), which were then applied to substrates including brass, steel, titanium, Al7075, copper, magnesium, and Al2024. Mechanical characterisation included hardness (H), reduced modulus (E), coating adhesion, and dissipated energy, calculated from the area of the load–unload hysteresis loop. Each coating–substrate combination underwent 36 nanoindentation tests, and adhesion was evaluated by pull-off tests. The initial results showed a poor correlation between adhesion and conventional mechanical properties (χ^2^ of 17.1 for hardness and 16.2 for modulus, both with R^2^ < 0.24). In contrast, the dissipated energy showed an excellent correlation with adhesion (χ^2^ = 0.52, R^2^ = 0.92), suggesting that dynamic deformation mechanisms better describe bonding. This introduces a new perspective to predict and optimise cold-spray adhesion in industrial applications.

## 1. Introduction

Adhesion refers to the phenomenon in which attractive forces between the substrate and the coating material create a durable bond. These forces arise from intermolecular interactions, including hydrogen bonds, van der Waals forces, and electrostatic interactions. Mechanical joints are of critical importance, as they significantly influence adhesion strength in many cases [1].

The value of adhesion forces is influenced by factors such as surface preparation and cleaning, surface roughness and topography, surface energy of the materials, and polarity of the molecules [2,3]. Naturally, in addition to adhesion, maintaining the integrity of the coating itself is also crucial. The measure of a coating’s integrity is the value of the cohesion forces. Cohesion is the phenomenon of attraction between molecules of the same substance, which ensures that the material retains its structural integrity. In cold-spray (CS) processes, numerous factors influence the value of adhesion. In their work, Prashar and Vasudev discussed methods for evaluating adhesion and identified the factors that affect its magnitude [4]. These factors include gas temperature, gas type, type of coating material, grain size and shape, and degree of grain oxidation. Furthermore, the properties of the substrate, such as hardness, temperature, and surface roughness, play an important role.

**Figure 1:**
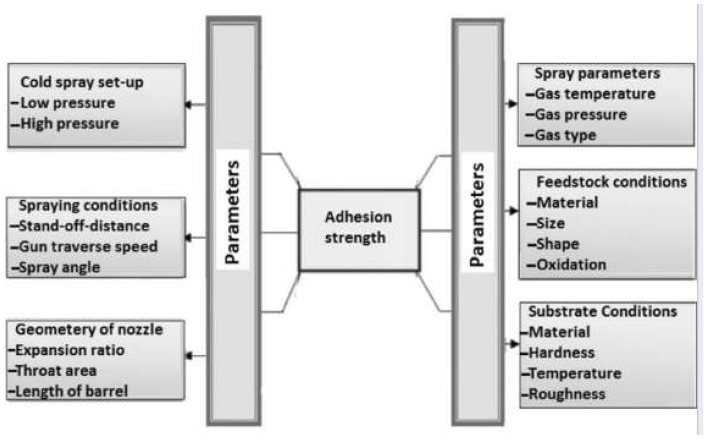
Selected process parameters in cold spray deposition that influence coating adhesion [8].

Figure 1 illustrates the categorisation of cold spray systems and the associated control parameters that govern the coating process. An innovative coating application method is the thermal spray technique, specifically cold spray, in which the coating material is applied to the substrate without altering the properties of the deposited material layer [5,6].

**Figure 2:**
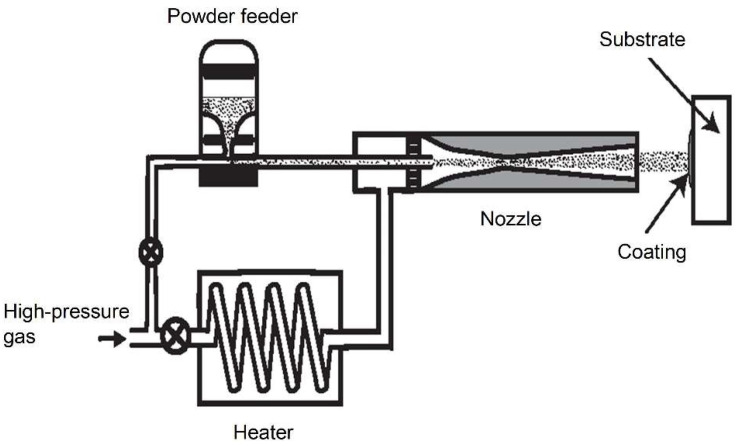
The concept of cold gas spraying, highlighting the key components of the spraying system, is presented.

The essence of the cold spray process is to give the coating material particles high kinetic energy because, after passing through a convergent–divergent nozzle, they hit the substrate and create a coating. Effective deposition requires achieving a threshold particle velocity, depending on the type of material, ranging from 300 to 1200 m/s. This velocity is achieved due to a carrier gas with a pressure of 0.5 to 15 MPa and a temperature lower than the powder melting point (usually 0 to 800 °C). Air, nitrogen, or helium are the most often used working gases. Figure 2 shows a schematic representation of the trajectory of a coating particle carried by the process gas.

Powder with a grain size of 5–150 μm is fed into the gas stream. In the initial phase of spraying, the surface is activated, oxides are removed, and the substrate is cratered. The coating then forms as a result of mechanical blocking, clogging, and adiabatic shearing [7,8].

The phenomenon of adiabatic shear instability is a local loss of material strength, which changes the deformation mechanism from plastic to viscous. In this respect, the size and shape of the particles are important; light and small particles are preferred, which more easily reach the required velocity. Too large particles have a larger surface area, which increases the risk of oxidation and is favourable for coating adhesion. A uniform particle size distribution facilitates the determination of the critical velocity and improves the quality of the coating. The key element of the system is the de Laval nozzle.

The deposited coatings serve various functions that enhance substrate properties. Functional coatings involve the application of a different material to provide features such as corrosion and wear resistance, electrical conductivity, or reduced viscosity. In part remanufacturing, a similar material is deposited to repair defects from wear, corrosion, or production flaws, restoring the original shape and function. Cold spray also enables additive manufacturing by creating thick layers (up to several centimetres), allowing near-net-shaped parts, and reducing post-processing needs.

Cold spraying, a technique introduced by Papiryn approximately 30 years ago, has become increasingly recognised, although it is still regarded as innovative in some industries [9]. In recent years, the cold-spray process has garnered significant attention because of its ability to produce dense, thick metal deposits while maintaining the purity of the sprayed powders. This is achieved without inducing phase transitions in the material, and there is no formation of new phases or oxidation of the coating material during the process. As a result, the process preserves the integrity of the properties of the material, making it highly advantageous for various applications.

The benefits of cold spray technology have become widely recognised, particularly in fields such as aerospace, biomedicine, and energy. Its ability to deposit high-quality coatings without an excessive thermal impact on both the coating and substrate materials has made it a valuable technique for producing durable and high-performance components.

The mechanical characteristics of titanium coatings applied by cold gas spraying, limited to hardness, modulus of elasticity, or adhesion, do not always fully reflect the properties of the coating. The perspective of analysing hysteresis in connection with adhesion is interesting, and its inclusion in the description of material properties makes sense, especially when we want to predict the effects of adhesion in dynamic and high-pressure processes, such as cold spray.

The primary objective of this study is to investigate whether the energy dissipated during nanoindentation, quantified as the area enclosed by the loading–unloading hysteresis loop, can serve as a meaningful indicator of bonding strength in cold-sprayed coatings [10,11]. Given the complex and high-velocity nature of particle–substrate interactions in cold spray deposition, this work aims to explore the extent to which quasi-static mechanical parameters—namely, hardness, elastic modulus, and dissipated energy—correlate with adhesion performance under fixed spraying conditions. By establishing such correlations, we seek to provide a simplified, laboratory-accessible framework to support process parameter selection and material compatibility assessment in cold spray technology. The Ti–Ti system serves as a reference configuration for optimising deposition conditions, which are then applied to other metallic substrates to assess their influence on coating adhesion and deformation behaviour.

## 2. Materials and Methods

The experiment focused on studying adhesion in relation to the mechanical properties of both the coating material and the substrate, while taking into account the values of the spray parameters. The shape and size of the Ti coating material particles were consistent in all cases.

### 2.1. Coatings and Substrate Types

**Figure 3:**
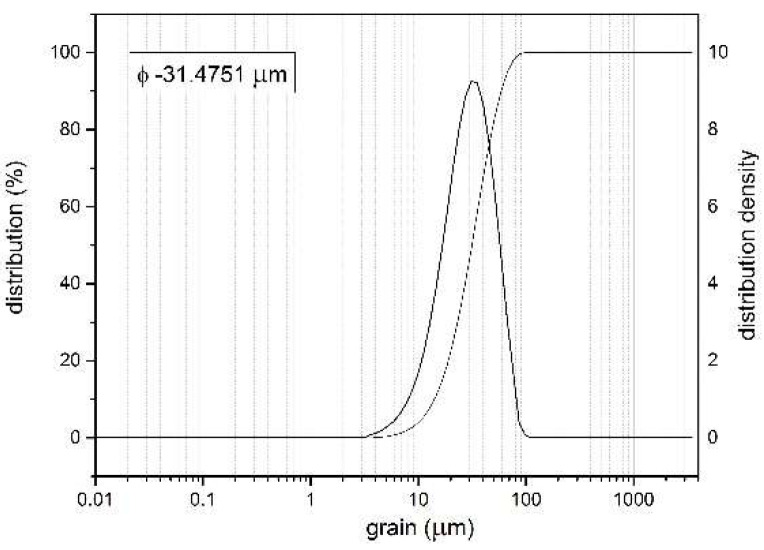
Particle Size Distribution of Titanium Powder.

Titanium powder with a particle size distribution shown in Figure 3 was selected as the coating material. For the spraying process, titanium particles with a granulometry range of 20–70 µm were chosen. The average particle size was approximately 31.5 µm. The Ti particles were approximately aspherical in shape. Commercially pure titanium powder (99 wt.% Ti) was used as feedstock in this study by Kamb Import–Export (Warsaw, Poland).

Seven metal plates were selected as substrates for the application of the titanium coating. Table 1 presents a detailed list of the materials used for the substrates, including their corresponding elastic modulus (E), which is a critical parameter in evaluating the mechanical properties of the substrate material. Young’s modulus provides insight into the stiffness of the material, which plays an important role in determining the behaviour of the coating during the deposition process and under operating conditions. These substrates were rectangular plates, each measuring 25 × 3 × 0.5 cm, providing an adequate surface area for uniform coating deposition. To ensure optimal adhesion between the coating and the substrate, we subjected the metal plates to surface preparation processes before coating. Specifically, the substrates were sandblasted to increase surface roughness, which is crucial for improving the mechanical interlocking between the coating and the substrate. In addition, plates were degreased to remove any oils, contaminants, or residues that could interfere with the coating process and adhesion quality.

To ensure the accuracy and reliability of the experimental results, all measurements were performed using a single consistent machine setup. This approach minimised the variability of the testing conditions, contributing to the precision of the data obtained and making the results more comparable between tests.

### 2.2. Research Equipment and Methods

Impact Innovations’ 5/8 industrial system was used to apply the coating, allowing the carrier gas to be heated to 800 °C. The parameters controlled in the process were temperature T, pressure p, spray head travel speed V, and head-to-substrate distance d. The head was held and controlled by the Fanuc M-20iA robot arm (FANUC Corporation, Oshino-mura, Yamanashi, Japan), ensuring that the V and d values were maintained over time. Nitrogen was used as a carrier gas in the application process, the temperature and pressure of which were controlled. An important element of the spray system was the use of a de Laval spray nozzle. The spraying conditions were determined using the G. Taguchi statistical method with the L9 plan [12,13].

### 2.3. G. Taguchi Industrial Statistics for Cold Gas Spray Applications

The Taguchi method is a structured approach to experimental design that aims to optimise processes and create high-quality systems. Reduce the number of experiments needed while maintaining precision and consistency. This approach, based on factorial design, uses an orthogonal array, a set of experiments performed under different conditions to assess and optimise the selected factors (variables). The Taguchi statistical design method significantly reduces the number of experiments while maintaining analytical effectiveness. For four control factors, each with three possible levels (resulting in 3^4^ = 81 total combinations), using a Taguchi orthogonal array L9 reduces the number of experimental configurations required to only nine. Known for its simplicity, effectiveness, and reliability, the Taguchi method is widely used in optimisation tasks [14].

The Taguchi design uses a loss function, which is transformed into a signal-to-noise ratio (S/N) to assess the deviation between the experimental results and the desired targets. The S/N ratio is calculated as the ratio of the mean response to its standard deviation, serving as a tool to pinpoint the optimal settings for each factor to improve performance. In the S/N analysis, performance characteristics are classified into three categories: lower-the-better, higher-the-better, and nominal-the-better. Furthermore, statistical analysis, often through analysis of variance (ANOVA), is used to identify the most significant variables. When the Taguchi design is combined with ANOVA, a powerful method is achieved to determine the optimal process conditions. The S/N ratio represents the relationship between the mean (signal) and the standard deviation (noise) and is influenced by the quality characteristics of the product or process being optimised. Commonly used S/N ratios include nominal-is-best (NB), lower-the-better (LB), and higher-the-better (HB).

In the experiment carried out, the HB procedure was chosen because of the goal of achieving the highest possible adhesion value of the coating to the substrate.

The S/N function takes the following form:

(1) SN=η=−10log101n ∑i=1n1yi2

where: y is the value measured in each repetition of i = 1, 2, ….

The formula for the signal-to-noise (S/N) ratio was proposed by G. Taguchi [14].

For each parameter (e.g., T), we calculate the difference between the maximum η value and the mean η value obtained in the experiment for all controlled quantities. This difference is a measure of the sensitivity of this parameter to changes in the experiment. The larger this difference, the more influential a given parameter level is on the final result.

(2) $$∆\eta_{T}=\eta_{max,T}-\bar{\eta_{T}}\;$$

where:

$\bar{\eta_{T}}$—the average value of *η*_*T*_ obtained from experimental measurements (hardness, Young’s modulus, adhesion) at a fixed parameter T,

$\eta_{max,T}$—the maximum η value observed for temperature T.

A larger Δ*η*_T_ indicates greater sensitivity of the process outcome to changes in *T*. Consequently, the parameter value T is updated according to the following:

(3) $$T_{new}=T_{old}+k\cdot \frac{{∆\eta }_{T}}{\sum ∆\eta }$$

k—is a calibration factor (assumed to be 1 in this study),

$∆\eta_{T}$—defined by Equation (2),

∑∆η—sum of differences for all control parameters (T, p, V, d).

The selection of parameters was performed for a titanium coating on a titanium substrate. In the experiment, the controlled parameters and their value ranges were chosen, which are presented in Table 2.

The L9 orthogonal matrix was selected for statistical optimisation.

The control parameters are hardness and modulus of elasticity, and on this basis, we estimate the optimal values of the controlled parameters using the formula of Equation (3).

### 2.4. Testing of Hardness, Elasticity Modulus, and Adhesion

Nanoindentation tests were performed to determine the hardness (H) and elastic modulus (E) of the coatings. Measurements were performed using a NANOVEA nanoindenter equipped with a Berkovich diamond tip (NANOVEA, Irvine, CA, USA). A maximum indentation load of 20 mN was applied with both loading and unloading rates set at 40 mN/min. The instrument was calibrated prior to testing using a standard fused silica sample, following the manufacturer’s recommended procedure.

Indentations were made on cross-sectional samples embedded in epoxy resin and polished to a mirror finish using progressively finer diamond suspensions down to 0.25 μm. A total of 36 indentations were made for each coating, with measurements systematically spaced in the vicinity of the coating–substrate interface to capture variations in mechanical response due to microstructural heterogeneity. Care was taken to avoid visible pores or surface defects during indent placement, and outliers or tests with poor unloading curves were discarded. Hardness (H) and elastic modulus (E) were extracted from load-displacement data using the Oliver W.C. method [15].

**Figure 4:**
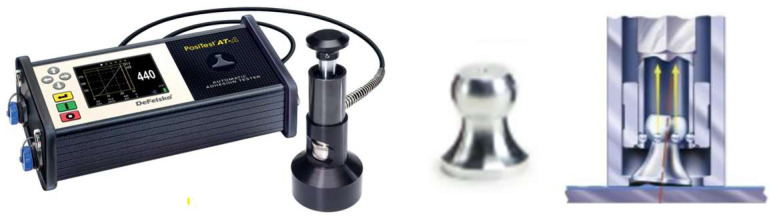
Vacuum pump and detachment mushroom holder.

The adhesion of the coatings was evaluated by measuring the vacuum generated beneath a mushroom-shaped probe that was attached to the coating surface—Figure 4. This method involves placing the probe on the coating, creating a sealed contact between the probe and the surface. Then, a vacuum is applied, and the resulting pressure difference is monitored. The degree of vacuum generated reflects the strength of the bond between the coating and the substrate. A higher vacuum indicates stronger adhesion, as it suggests that the coating is securely attached to the substrate, with minimal air gaps or separation. This technique provides valuable information on the quality and effectiveness of the adhesion properties of the coating.

The detachment mushroom holder was adhered to the coating and subsequently detached by creating a vacuum ranging from 0 to 50 MPa. The diameter of the mushrooms used was 15 mm. Each coating was tested three times, and the result assigned to the coating was considered the average value.

### 2.5. Mechanical Response

The mechanical properties of titanium coatings produced by cold-gas spraying are important in assessing their suitability for industrial or biomedical applications. Standard mechanical characterisation methods focus mainly on adhesion, cohesion, hardness, and modulus of elasticity. However, the elasticity and flexibility properties of titanium coatings, despite their fundamental importance for the structural integrity of the coating-substrate system, are rarely analysed through the prism of the hysteresis symmetry generated by the loading and unloading force [16,17,18]. The review of the literature shows that most studies in the area of cold gas spray technology are limited to determining basic mechanical parameters, not fully utilising the information contained in the hardness test data. For example, studies that use nanoindentation to analyse the mechanical properties of titanium coatings mainly focus on hardness and elastic modulus, omitting analysis of load–unloading curves [19]. Similarly, other works focus on the microstructure and basic mechanical properties without a detailed analysis of the elastoplastic response of the coatings [20].

**Figure 5:**
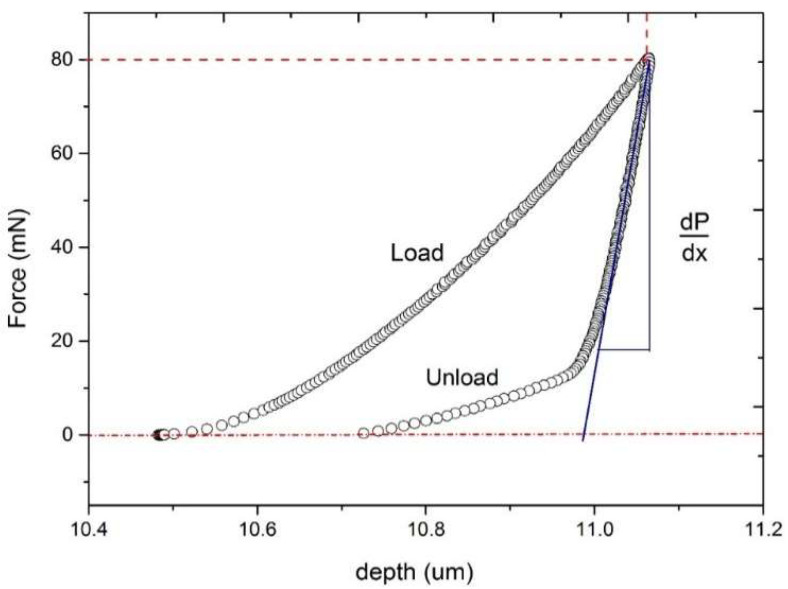
Typical indentation curves obtained during hardness testing using the instrumented indentation method.

Therefore, the study analysed the size of the area between the load and unload curves. Figure 5 shows the load and unload curve of a typical hardness test experiment.

The elastic modulus is calculated from the initial slope of the unload curve, with the subsequent portion of the curve being ignored. Some materials exhibit a very short unload curve, which reflects the almost unchanged indentation depth during indentation testing. In certain cases, the load curve reaches zero, indicating that the indentation in the material is significantly smaller than its maximum dimensions. This relationship suggests an alternative approach to plasticity, considering not only the shape of the curve but also the area enclosed by it. The area under the load curve represents the work that the indenter performs to achieve the applied load force. The area under the unload curve reflects the work performed by the material as it expels the indenter. The difference between the work performed by the indenter and the material is referred to as “workability” in this context.

The work required to press the indenter to a given depth is calculated using the following classic formula:

(4) $$W_{I}=\int_{0}^{l}F_{l}dx$$

where: 0, l are the limits of the displacement of the indenter under the influence of force F.

To quantify the energy dissipation during nanoindentation, we employed a direct approach based on the integral of the displacement curve. This method captures the actual mechanical work performed during loading and unloading, reflecting energy losses associated with irreversible deformation, microstructural friction, and hysteresis effects. This approach avoids the need for complex constitutive modelling, which would require detailed assumptions about material parameters not directly accessible from quasi-static indentation data. Although simplified, this method provides a robust and experimentally grounded metric for comparing dissipation behaviour across different coating–substrate systems and evaluating its correlation with adhesion strength [21].

Dissipated energy was defined as the difference in the work achieved by loading and unloading. This approach can provide a more comprehensive understanding of the mechanical behaviour of these coatings, which is crucial for their applications.

## 3. Results and Discussion

The experiment was divided into three parts. In the first case, the conditions for spraying a titanium coating on a titanium substrate were established. In the second step, under the specified spraying conditions, titanium coatings were applied to metal substrates with different mechanical properties (Table 1). The third stage was related to the determination of the adhesion value and the attempt to describe its dependence on the modulus E of elasticity.

The selection of parameters for the deposition of a titanium coating on a titanium substrate was carried out using industrial statistics based on the Genichi Taguchi method [14]. One of the main advantages of this method is the significant reduction in the number of experiments required, along with the flexibility to adjust the parameter values in real time. Four controlled parameters were chosen: temperature (T), pressure (p), velocity (V), and distance (d), with each parameter having three possible values. Without the application of the Taguchi method, the total number of combinations would be 3^4^ = 81, which is a substantial number of experiments. However, applying the Taguchi statistical method, the number of experiments was reduced to only 9. The experimental design and its details are presented in Table 3. This approach not only saves resources but also streamlines the process of determining the optimal conditions for coating deposition.

After parameter selection was completed, a verification experiment was performed to assess the effectiveness of the parameters chosen in controlling the spray process. In this phase, the experiment aimed to confirm whether the selected conditions would produce the desired coating properties, such as optimal adhesion.

**Figure 6:**
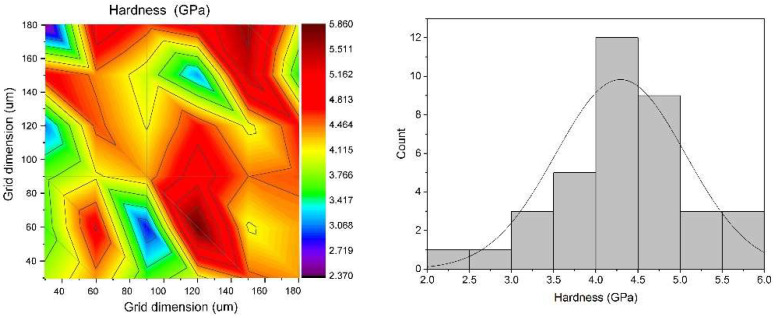
Hardness map and the corresponding histogram of the coating on a titanium substrate (T = 800 °C, p = 45 bar, d = 40 mm, V = 300 mm/s) obtained under experimental conditions defined in the Taguchi L9 design.

**Figure 7:**
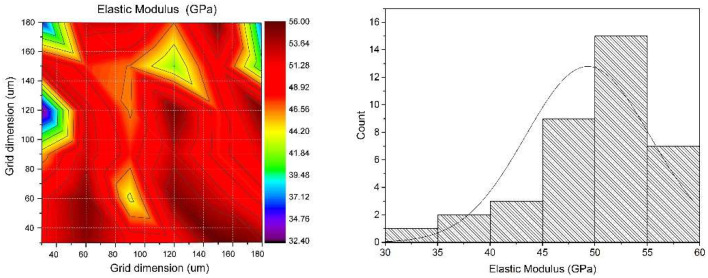
Map of the elastic modulus and the corresponding histogram for the coating on a magnesium substrate (T = 700 °C, p = 45 bar, d = 50 mm, V = 500 mm/s), obtained under the experimental conditions defined in the Taguchi L9 design.

After applying the titanium coating to the titanium according to the L9 plan (Table 3. Experimental plan according to G. Taguchi), the obtained coatings were tested by measuring H and E. Hardness H and the modulus of elasticity E were measured in cross sections using an indentation force of 20 mN. For each sample obtained, 36 H and E measurements were made. Based on these data, average values were calculated and used as a basis for further analysis. There is no theoretical basis to interpret the hardness variations observed in the hardness map as indicative of a heterogeneous material phase, since the phase transition temperature exceeds the temperatures applied in this study. Therefore, the observed hardness discrepancies are attributed solely to substrate inhomogeneities. Figure 6 and Figure 7 show the distribution map of hardness and modulus of elasticity for a selected sample from the L9 plan. The distance between the measurement points was approximately 20 μm, with an average depth of measurement indentations of 1–2 μm.

**Figure 8:**
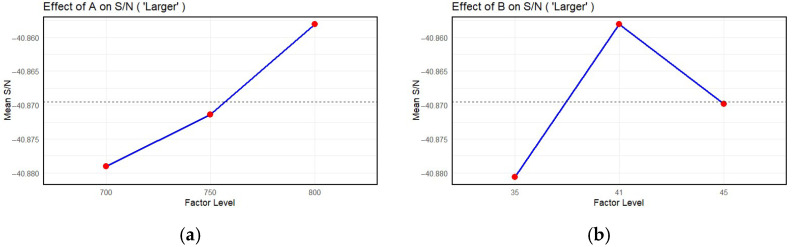

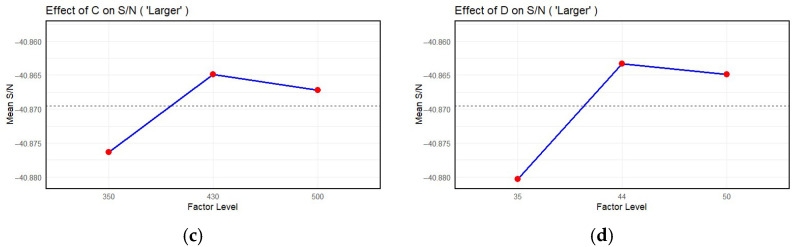
Taguchi analysis diagrams for coating performance optimisation: (**a**) temperature, (**b**) pressure, (**c**) speed, and (**d**) distance.

Figure 8 shows the analysis of the influence of the control parameters on the hardness values obtained. To identify the optimal process parameters, a Taguchi analysis was performed using the signal-to-noise (S/N) ratio as the performance metric, under the assumption that ‘larger is better’, meaning that higher values of the response variable are more desirable (Equation (3). The analysis was carried out for four factors and their corresponding diagrams (Figure 8a–d): temperature T, pressure p, velocity V, and distance d.

Temperature T: the S/N ratio increases steadily, with the highest value recorded at level 800. This trend suggests a strong positive correlation between the level of factor A and the performance of the process, indicating that increasing the value of this parameter contributes to improved response quality. Therefore, level 800 is the most favourable setting for factor A within the tested range. Figure 8b presents the influence of factor B on the S/N ratio. The mean S/N ratio reaches its maximum at the intermediate level of 41 bar, while the lower level (35) and the higher level (45) produce lower values. This suggests the presence of an optimal point for the factor p, beyond which the performance of the process may degrade. The effect of velocity V on the signal-to-noise ratio (S/N) shows an increase from 350 to 430, indicating that initially increasing this parameter has a beneficial effect. However, a slight decrease is observed when the level is increased further to 500. This behaviour implies a local optimum at level 430, beyond which further increases may lead to diminishing returns or even to a deterioration in performance. In the last case, there is a diagram that analyses the effect of distance d on the S/N ratio (Figure 8d). This diagram suggests that the best value of d is 44 mm.

Based on the G. Taguchi analysis, the optimal combination of parameter levels that maximises the response within the ‘bigger is better’ criterion, taking into account the influence of individual parameters on S/N using the relationship Equation (3), we obtain:

Taking the hardness into account as a control parameter, the following was obtained:

T_newH_ = 732.2 °C, p_newH_ = 32.5 bar, V_newH_ = 345.3 mm/s, d_newH_ = 35.6 mm.

Similar calculations were performed using the measurement of the control parameter E:

T_newE_ = 729.8 °C, p_newE_ = 33.4 bar, V_newE_ = 341.9 mm/s, d_newE_ = 35.3 mm.

In the process of selecting the controlled parameters for the deposition of the Ti coating on titanium, the values of the control parameters were selected as the average values obtained in the H and E optimisation.

T_new_ = 731.0 °C, p_new_ = 33.0 bar, V_new_ = 343.6 mm/s, d_new_ = 35.5 mm (5)

**Figure 9:**
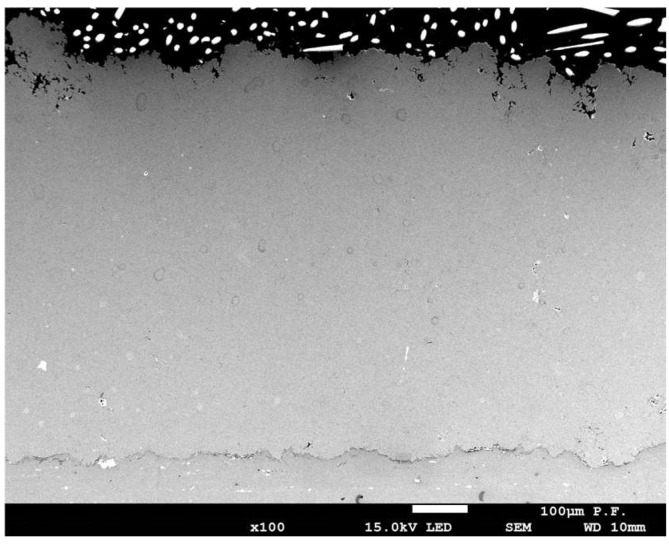
Cross-section of the Ti coating on a Ti substrate, obtained using process parameters determined through optimisation (T = 731.0 °C, p = 33.0 bar, V = 343.6 mm/s, d = 35.5 mm).

An experiment was conducted to verify the quality of the deposited coating and to confirm the optimal spray parameters. The resulting coating, whose cross-section is shown in Figure 9, corresponds to the titanium coating deposited on a titanium substrate (Ti–Ti system), which served as a reference configuration for the optimisation of the process. The spraying parameters determined for the Ti–Ti system were subsequently applied—without modification—to other metal substrates with different elastic moduli to investigate how the mechanical properties of the substrate influence coating adhesion under identical deposition conditions.

The cross-sectional micrograph reveals a distinct coating layer located at the upper part of the image (Figure 9). The interface between the coating and the substrate exhibits a degree of irregularity, which may suggest variations in the adhesion quality or effects related to the specific parameters of the cold-spray process. The substrate itself appears relatively homogeneous, with only minor contrast variations that may indicate subtle microstructural inhomogeneities. The surface of the coating demonstrates noticeable roughness and discontinuities, which are characteristic of coatings applied by solid-state deposition techniques such as cold spraying [22]. The titanium coating exhibits a certain degree of porosity and structural heterogeneity, which is typical of metallic coatings deposited using cold spray methods. The interface between the coating and the titanium substrate is clearly defined yet non-uniform, suggesting local differences in bonding quality, possibly arising from fluctuations in particle velocity or surface topography of the substrate prior to deposition [23].

With the values of the spray parameters determined in this way (Equation (4)), a titanium coating was applied to all substrates listed in Table 1.

Control measurements of hardness (H) and elastic modulus (E) were performed on cross-sectional samples.

**Figure 10:**
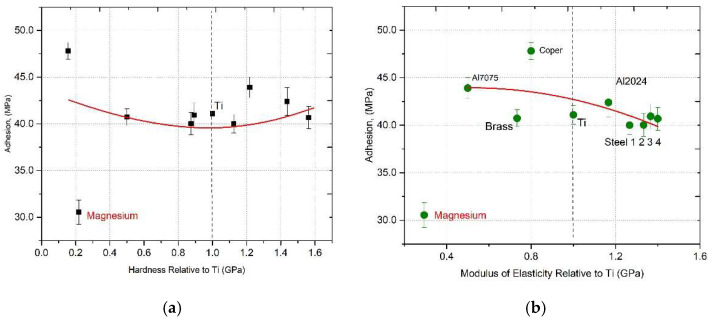
(**a**) Adhesion as a function of hardness (H); (**b**) Adhesion of the examined coatings as a function of elastic modulus (E).

The adhesion test for each coating was repeated three times, with the adhesion value taken as the average. The results were collected and presented graphically in Figure 10.

The horizontal axes of the graphs are scaled with respect to the values obtained for titanium, where both the hardness and modulus of elasticity for Ti were assigned a value of one.

In Figure 10a, the adhesion values are lowest for magnesium, highest for copper, and then decrease for steel. The relationship between elastic modulus and adhesion is expected to follow a similar trend. However, in this case, the adhesion of the steel differs significantly from the values observed for other substrates. This is due to the considerably higher hardness and elastic modulus of the titanium coating compared to that of the steel substrate. This difference arises from the insufficient kinetic energy of the particles impacting the steel surface, which cannot induce the surface deformation necessary for mechanical interlocking. The preparation of the sample surface for spraying may have a significant effect on adhesion. All samples were prepared in the same way and sandblasted with electrocorundum. As a result of the differences in H and E of the substrates used, the roughness between the individual samples could differ significantly. Focussing on the analysis of the adhesion values in the cases discussed, a second-order function fit was carried out (y = ax^2^ + bx + c), and the fit of the function to the experimental points was evaluated using Reduced Chi-Squared (χ^2^) and R-Squared (R^2^), also known as the coefficient of determination. To analyse the relationship between dissipated energy and measured adhesion strength, linear and second-order polynomial (quadratic) regression models were considered. Although a linear model might suggest a simple proportional trend, preliminary analysis revealed systematic deviations in the data from a straight line. In contrast, the second-order polynomial model demonstrated a better fit, with a coefficient of determination (R^2^) of approximately 0.92, compared to ~0.75 for the linear model. This improvement indicates a better ability to capture the curvature observed in the data distribution, suggesting a nonlinear dependence of adhesion strength on the mechanical response of the coating–substrate system. Additionally, Spearman’s rank correlation analysis was conducted to assess the monotonicity of observed trends. The results confirmed that the relationship between dissipated energy and adhesion strength remains statistically consistent in both individual and averaged steel data, further supporting the validity of the fitted quadratic model despite the limited sample size.

For adhesion as a function of hardness, the following was obtained: χ^2^ = 17.1062, R^2^ = 0.2369.

For adhesion as a function of elastic modulus χ^2^ = 16.1937, R^2^ < 00.2369 (df − 9, α = 0.05).

The fit of functions to experimental points is not satisfactory.

Studies have shown that the adhesion efficiency of the cold-spray process depends on the ability of the particles to deform and form metallic bonds with the substrate. In the case of hard particles, such as titanium, hitting a steel substrate, there may not be sufficient deformation of the substrate surface, which limits the possibility of mechanical anchoring of the coating. In their study, Yin et al. analysed how substrate hardness and spray angle affect the behaviour of titanium particles during the cold-spray process. Differences in hardness between the coating and the substrate were found to affect the adhesion mechanism, since the kinetic energy of the particles may be insufficient to induce the necessary plastic deformation of the substrate surface, which limits the possibility of mechanical anchoring of the coating [24]. Assadi et al. analysed the adhesion mechanisms in the cold-spray process, emphasising the importance of plastic deformation of the particles and the substrate to achieve a strong bond. They noted that differences in hardness and elastic modulus between the coating and the substrate can affect the adhesion efficiency [25]. Similar results and conclusions are found in the work of Villafuerte [26]. Although a small number of materials are considered in the calculations presented in Figure 10, it can be concluded that there is a linear relationship between adhesion and hardness and the elastic modulus of materials with similar mechanical properties.

Based on the analysis of Figure 10b, it appears that the elastic modulus may not be the most appropriate parameter to describe the adhesion behaviour and could potentially be replaced by a more representative property of the material. Given the poor correlation between traditional mechanical parameters and adhesion, this study explores hysteresis-based parameters that capture the dynamic deformation behaviour during loading–unloading cycles, termed dissipated energy.

The literature review indicates that the elasticity and flexibility characteristics of titanium coatings, despite their fundamental importance for the structural integrity behaviour of the coating-substrate system, are rarely analysed through the prism of hysteresis symmetry. Most studies conducted in the area of cold gas spraying technology are limited to determining basic mechanical parameters and not fully using the information contained in the hysteresis characteristics of the material. This phenomenon is particularly important in the context of evaluating the boundary zone between the titanium coating and the titanium substrate, where mechanical interactions determine the durability of the connection.

The foundational work in the methodology for analysing load–unloading curves is the work of Oliver and Pharr [27], which, although not directly related to cold gas spray technology, provides a theoretical basis for the interpretation of hysteresis phenomena in nanoindentation studies. Cavaliere et al. presented more focused studies that analysed hysteresis loops during fatigue tests of cold gas-sprayed coatings, showing a correlation between hysteresis asymmetry and fatigue life of coatings [28].

Previous studies using hysteresis analysis in the context of cold gas-sprayed coatings focus mainly on three aspects: assessing the range of elastic and plastic deformations, determining the energy dissipated during load–unload cycles, and determining residual stresses [29,30,31]. However, the full potential of hysteresis symmetry analysis remains untapped, especially with regard to the characterisation of the coating-substrate interface. This literature gap motivated the current investigation into the hysteresis-based characterisation of coating adhesion. Due to the unsatisfactory description of adhesion in relation to the modulus of elasticity, attention was focused on the measurement related to the displacement of the indenter. The load and unload curves form half of the hysteresis, and the surface area between them determines the work performed on the sample when the indenter is pressed and retracted. This approach can provide new and valuable information on the mechanisms of adhesion and deformation in coating-substrate systems, complementing studies that focus only on the modulus of elasticity and hardness. Table 4 summarises the test results according to the procedure described in Section 2.

**Figure 11:**
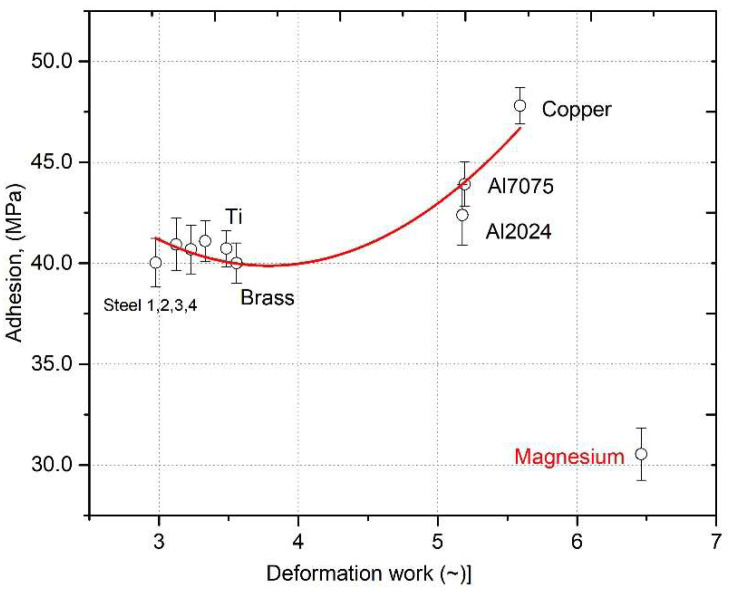
Adhesion of titanium coatings as a function of dissipated energy on various metallic substrates.

The measurement results are shown in Figure 11. The results for steels 1–4 and brass are similar. Adhesion clearly increases for Al and Copper alloys. The magnesium results were rejected due to very low adhesion caused by a too large difference in H and E between the substrate and the coating [32,33]. Surprisingly, the adhesion value for copper is quite high compared to that of Ti on the Ti coating.

By excluding the adhesion results obtained for the magnesium substrate, the fitting parameters were obtained: χ^2^ = 0.5211 for six degrees of freedom and the significance level α = 0.05; the critical value is approximately 12.59. Our χ^2^ value is much lower, which confirms that the model fits the data well, R^2^ = 0.9247.

## 4. Conclusions

This study examined the adhesion of titanium coatings deposited by cold spraying onto various metallic substrates and analysed their relationship with mechanical properties. The Taguchi method (L9 design) was successfully employed to optimise the spray parameters, reducing the experimental runs from 81 to 9 and identifying optimal conditions: temperature 731.0 °C, pressure 33.0 bar, spray velocity 343.6 mm/s, and stand-off distance 35.5 mm. The spray parameters (temperature, pressure, outlet velocity, and stand-off distance) were fixed on the basis of optimisation for the Ti–Ti system, to isolate the influence of substrate mechanical properties on coating adhesion.

Initial analysis showed that traditional mechanical parameters, hardness and elastic modulus, correlate poorly with coating adhesion, limiting their utility as predictive indicators. On the contrary, dissipated energy, defined as the energy lost during loading–unloading nanoindentation cycles and capturing the hysteresis behaviour of the material, exhibited a much stronger correlation with adhesion.

Importantly, excluding data for the magnesium substrate, the fitting of the model improved markedly: a Chi-Squared (χ^2^) value of 0.5211 with six degrees of freedom—well below the critical threshold of 12.59 at α = 0.05—indicates an excellent fit, supported by a high coefficient of determination (R^2^ = 0.9247). This confirms that dissipated energy is a robust parameter for predicting adhesion across most substrates studied.

These findings suggest that adhesion in cold-sprayed titanium coatings depends not only on intrinsic material properties but also on the dynamic deformation response of the coating-substrate system. Incorporating dissipated energy as a key descriptor offers a more comprehensive and reliable approach for predicting and optimising adhesion, with significant implications for industrial cold-spray processes and coating design. Future research should explore the applicability of dissipated energy for other coating materials and deposition techniques.

## Figures and Tables

**Table 1:** Summary of substrate materials used in the study.

Substrate	E [GPa]
Al2024	175.0 ± 11.0
Al7075	155.0 ± 9.0
Brass	110.0 ± 7.5
Copper	120.0 ± 8.0
Magnesium	40.0 ± 0.1
Steel1	210.0 ± 7.6
Steel2	190.0 ± 5.5
Steel3	200.0 ± 12.1
Steel4	205.0 ± 13.4
Titanium	150.0 ± 8.0

**Table 2:** Set of cold gas spray parameters.

T [°C]			p [bar]			d [mm]			V [mm/s]		
700	750	800	30	37	45	30	40	50	300	400	500

**Table 3:** Experimental design based on the G. Taguchi method: Orthogonal Array L9 representing configurations for four control factors at three levels each.

Trial No.	Input Parameters			
	T [°C]	p [bar]	d [mm]	V [mm/s]
1.	700	30	30	300
2.	700	37	40	400
3.	700	45	50	500
4.	750	30	40	500
5.	750	37	50	300
6.	750	45	30	400
7.	800	30	50	400
8.	800	37	30	500
9.	800	45	40	300

**Table 4:** Comparison of Adhesion and Dissipated Energy Values for Ti Coating on Various Substrates.

Substrate	E (GPa)	Adhesion (MPa)	Dissipated Energy (J)
Al2024	175.0 ± 11.0	42.4 ± 1.5	5.2 ± 0.2
Al7075	155.0 ± 9.0	43.9 ± 1.1	5.2 ± 0.2
Brass	110.0 ± 7.5	40.7 ± 0.9	3.5 ± 0.2
Copper	120.0 ± 8.0	47.8 ± 0.9	5.6 ± 0.2
Magnesium	44.0 ± 0.1	30.5 ± 1.3	6.5 ± 0.2
Steel1	210.0 ± 7.6	40.7 ± 1.2	3.2 ± 0.2
Steel2	190.0 ± 5.5	40.0 ± 1.0	3.6 ± 0.2
Steel3	200.0 ± 12.1	40.0 ± 1.2	3.0 ± 0.2
Steel4	205.0 ± 13.4	41.0 ± 1.3	3.1 ± 0.2
Titanium	150.0 ± 8.0	41.1 ± 1.0	3.3 ± 0.2

## Data Availability

The original contributions presented in this study are included in the article. Further inquiries can be directed to the corresponding author.
